# A Case of Sympathetic Ophthalmia After Corneal Perforation

**DOI:** 10.7759/cureus.74579

**Published:** 2024-11-27

**Authors:** Koichi Taguchi, Hiroshi Toshida, Saya Kimura, Chikako Suto

**Affiliations:** 1 Ophthalmology, Tokyo Women’s Medical University Adachi Medical Center, Tokyo, JPN; 2 Ophthalmology, Juntendo University Shizuoka Hospital, Shizuoka, JPN

**Keywords:** corneal perforation, corneal ulcer, pulse steroid therapy, sympathetic ophthalmia, therapeutic corneal transplantation

## Abstract

We report a case of sympathetic ophthalmia that developed in the fellow eye following therapeutic corneal transplantation and amniotic membrane transplantation for corneal perforation caused by corneal ulceration. A 62-year-old man presented with discharge, lacrimation, and decreased visual acuity in the left eye. He was diagnosed with a corneal ulcer and treated with antimicrobial agents, but corneal epithelial erosion persisted, leading to nontraumatic corneal perforation. The patient underwent therapeutic lamellar keratoplasty and amniotic membrane transplantation, but corneal epithelial erosion remained. Subsequently, conjunctival flap surgery and tarsorrhaphy were performed. Soon after, sympathetic ophthalmia developed in the fellow eye, and pulse steroid therapy was initiated. Although recurrence occurred during steroid tapering, a second pulse steroid therapy with a gradual dosage reduction over about one year successfully prevented further recurrence. The patient completed steroid therapy with a good visual prognosis. This case highlights the development of sympathetic ophthalmia in the fellow eye after multiple surgeries for nontraumatic corneal perforation, and immediate steroid therapy proved effective.

## Introduction

Sympathetic ophthalmia is a granulomatous uveitis that occurs in both eyes after an ocular perforating injury or intraocular surgery [1-3]. It is an autoimmune disease against melanocytes, and the clinical findings are similar to those of Vogt-Koyanagi-Harada (VKH) disease [2,3]. In VKH, the immune response occurs when the body becomes infected with a virus, whereas sympathetic ophthalmia is triggered when the uveal tissue is invaded during intraocular surgery or in an ocular perforating injury. In both conditions, T cells specific to antigens such as tyrosinase expressed in activated melanocytes cause inflammation in melanocyte-containing tissues; this is considered a common pathology. According to the international diagnostic criteria for VKH, “the diagnosis requires the exclusion of a history of ocular trauma and intraocular surgery” [4]. Several case reports have been published on VKH, but the literature contains few reports on sympathetic ophthalmia due to nontraumatic corneal perforation.

Here, we report a case of sympathetic ophthalmia in the fellow eye that developed after therapeutic corneal transplantation and amniotic membrane transplantation for corneal perforation due to corneal ulceration.

## Case presentation

The patient was a 62-year-old man. Two months prior to the initial visit, he had developed a chalazion in the left eyelid, which spontaneously ruptured the following month; however, he did not seek ophthalmic care at that time. Although discharge and tearing persisted afterward, he chose to monitor the condition on his own without consulting a doctor. Approximately three weeks later, he experienced sudden pain in the eye and was examined at a nearby doctor’s office the following day. He was diagnosed with a left corneal ulcer and was referred to our department on the same day for further examination and treatment. He had no history of hypertension, diabetes, ocular trauma, or ophthalmic surgery, and while the mechanism of corneal ulceration onset was unclear, a possible link to the chalazion two months earlier could not be ruled out.

**Figure 1:**
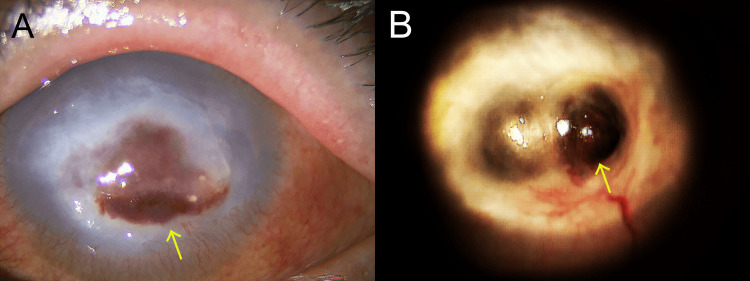
Corneal findings of the left eye (A) Corneal opacity and central corneal blood staining (arrow) were noted at initial presentation. (B) The central cornea was perforated at its thinnest point (arrow), with a flat anterior chamber and iris incarceration at the site of the perforation.

At the first visit, corrected visual acuity was 20/16 in the right eye and light perception (uncorrectable) in the left eye. At the first medical examination, in the right eye, no marked changes were seen in the anterior eye segment, optical media, or fundus, but in the left eye, significant subconjunctival hyperemia was observed, together with opacity over the entire cornea and central corneal blood staining. Although the anterior chamber was visible in the peripheral region, the presence of floating cells could not be confirmed because of the central corneal opacity, and the fundus was invisible (Figure 1A). Seidel’s sign was not present in the left eye, and intraocular pressure (IOP) was 21 mmHg on the right side and unmeasurable on the left side. Ultrasonography of the left eye did not show high reflective areas in the vitreous cavity, suggesting that the inflammation had not extended into the eye.

Bacterial keratitis was suspected in the left eye, and corneal scraping cultures were examined. Moxifloxacin hydrochloride ophthalmic solution (Vegamox^®^, Novartis Pharma, Tokyo, Japan), cefmenoxime hydrochloride ophthalmic solution (Bestron^®^, Senju Pharmaceutical Co., Ltd., Osaka, Japan), and tobramycin ophthalmic solution (Tobracin®, Nitto Medic Co. Ltd., Toyama, Japan) were administered to the eye six times a day for the first month and four times a day thereafter, and ofloxacin ophthalmic ointment (Tarivid^®^, Santen Pharmaceutical, Co., Ltd., Osaka, Japan) was administered four times daily. Concurrently, levofloxacin hydrate (Levofloxacin Bag for I.V. Infusion 500 mg “TAKEDA TEVA”^®^, Takeda Pharmaceutical, Co., Ltd., Osaka, Japan) was administered for 1 week. The results of the bacterial culture test obtained during this period were negative, and because no improvement was observed, imipenem/cilastatin sodium (2 mg; Tienam^®^, MSD, Rahway, NJ, USA) was subsequently administered for 5 days. The signs of infection disappeared with this treatment, but corneal epithelial defects persisted.

During outpatient follow-up on disease day 41, thinning and anterior protrusion were noted at the center of the cornea of the left eye and persistent corneal epithelial defects were observed, and it was determined that corneal perforation was highly likely to occur; therefore, therapeutic lamellar keratoplasty (LKP) was planned as an emergency procedure. After arrangement of a donor cornea, on the day when the emergency operation was scheduled (disease day 46), perforation of the central thin portion of the left cornea, flat anterior chamber, and iris incarceration into the site of the corneal perforation were observed (Figure 1B). LKP was performed on the same day with a cryopreserved cornea. The anterior chamber depth was normalized, and the iris was repositioned; the operation was completed upon confirming that there was no leakage of the anterior chamber fluid from the graft suture site. 

**Figure 2:**
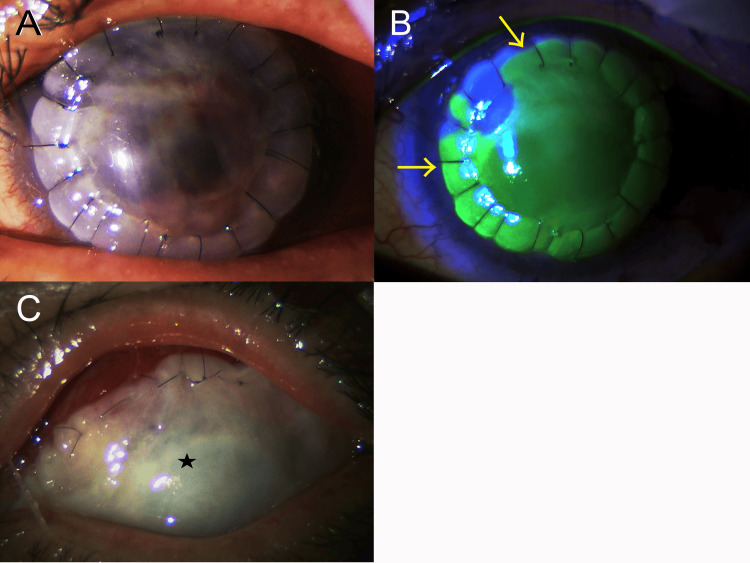
Postoperative corneal findings following surgical treatment for corneal perforation (A) Cornea on day six after lamellar keratoplasty for covering the perforation site. (B) Corneal epithelial defects observed around almost the entire circumference (arrows) 1 month after the initial lamellar keratoplasty. (C) Entire cornea covered by the amniotic membrane (asterisk).

Figure 2A shows a photograph of the anterior eye segment on postoperative day 6.

Although the site of corneal perforation was covered, corneal epithelial defects were observed immediately postoperatively, so sodium hyaluronate ophthalmic solution, rebamipide UD ophthalmic solution, and ofloxacin ophthalmic ointment were administered four times a day; however, the patient resisted treatment, and 1 month postoperatively, corneal epithelial defects were still present (Figure 2B).

Therefore, on disease day 81 (postoperative day 35), amniotic membrane transplantation was performed (Figure 2C). One month after the operation, after the amniotic membrane had dissolved, corneal epithelial defects in the upper cornea were observed again.

For this reason, the administration of the above-mentioned ophthalmic solutions and ofloxacin ophthalmic ointment were continued.

**Figure 3:**
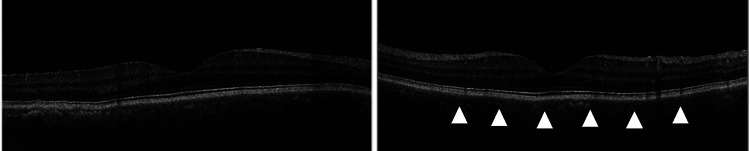
Optical coherence tomography findings at initial presentation At the initial presentation of sympathetic ophthalmia, mild choroidal folds were observed, indicated by a triangle marker with its tip pointing to the specific location.

On disease day 180, the corrected visual acuity of the right eye was 20/20, but keratic precipitates, inflammation in the anterior chamber, and inflammatory cells in the anterior vitreous were observed under a slit-lamp microscope, and the right IOP was high at 31 mmHg. Optical coherence tomography (OCT) of the fundus with a mydriatic pupil revealed mild choroidal folds, but no serous retinal detachment was observed (Figure 3).

Fluorescein angiography (FA) of the fundus revealed no multiple fluorescent leaking spots or optic nerve head fluorescence, and indocyanine green angiography of the fundus also revealed no hypofluorescent spots. No accompanying symptoms of VKH, such as headache, paresthesia in the scalp or hair, hearing loss, or tinnitus, were present. Blood tests showed no inflammatory responses or increases in the levels of immunoglobulin and complement activity, and a cerebrospinal fluid examination showed cytosis, with a cell count of 8/μl (Table 1). No abnormalities were observed on the chest X-ray. Based on these findings and the patient’s medical history, sympathetic ophthalmia was suspected. Although the diagnosis was determined to be an indication for pulse steroid therapy, the patient was treated by a drip infusion of 500 mg/day methylprednisolone for 3 days because no decrease in visual acuity was observed and because of the patient’s relatively old age and the absence of serous retinal detachment on OCT, among other things. In addition, treatment with betamethasone phosphate 0.1% eye drops (Rinderon^®^ 0.1%, Shionogi Pharmaceutical, Osaka, Japan) was initiated four times a day and Carteolol 2% eye drops (Mikelan^®^ LA, Otsuka Pharmaceutical, Tokyo, Japan) was once a day.

**Table 1:** Blood and cerebrospinal fluid test results for diagnosis of sympathetic ophthalmia Based on the blood test results on day 180, infectious and other inflammatory ocular diseases were ruled out. Considering the cerebrospinal fluid test results and the history of corneal perforation, a diagnosis of sympathetic ophthalmia was made. Abnormal values are highlighted in bold italics.

Blood tests	Day 1	Day 180	Day 406	Reference range
White blood cell count (×10^3^/µL)	9700	12000	8900	3300-8600
Neutrophils (%)	81	61.9		41.0-68.0
Eosinophils (%)	0	2.3		0.0-7.0
Basophils (%)	0.3	0.8		0.0-3.0
Monocytes (%)	4.2	6		2.0-8.0
Lymphocytes (%)	14.4	29		25.0-45.0
C-reactive protein (mg/dL)	0.03	0.03	0.04	≤0.14
Erythrocyte sedimentation rate (mm/h)	2	2	2	2.0-10.0
Angiotensin-converting enzyme (U/L)	11.8	11.9		8.3-21.4
Rheumatoid factor (IU/mL)	<5.0	<5.0		≤15.0
Immunogloblin G		837.9		861.0-1747.0
Immunogloblin A		62.9		93.0-393.0
Immunogloblin M		98.4		33.0-183.0
Complement C3		70.3		73.0-138.0
Complement C4		19.5		11.0-31.0
Treponema pallidum hemagglutination assay	Negative	Negative		
Rapid plasma reagin	Negative	Negative		
Cerebrospinal fluid (CSF) test				
CSF count		8		
Monocytes (%)		25		
Neutrophils (%)		0		

On disease day 183, methylprednisolone was started at 50 mg/day as a steroid after-treatment, and during follow-up, anterior chamber inflammation and keratic precipitates improved rapidly. On disease day 189, conjunctival flap surgery and tarsorrhaphy were performed for persistent corneal epithelial defects in the left eye.

**Figure 4:**
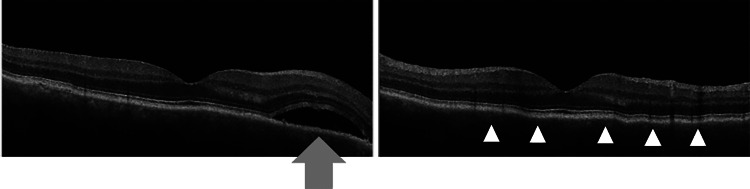
Optical coherence tomography findings during prednisolone treatment Serous retinal detachment (indicated by an arrow) and choroidal folds (indicated by a triangle) were observed in the parafoveal region. These findings resolved following a sub-Tenon injection of triamcinolone acetonide.

On disease day 198, during administration of 40 mg/day methylprednisolone, serous retinal detachment and choroidal folds were observed in the right eye (Figure 4), and corrected visual acuity of the right eye decreased to 20/25.

Recurrence of sympathetic ophthalmia was suspected, and a sub-Tenon injection of triamcinolone acetonide was performed; as a result, the serous retinal detachment and choroidal folds disappeared, and the corrected visual acuity of the right eye recovered to 120/20. Subsequently, the dosage of oral methylprednisolone was gradually tapered by 5 mg every 1 to 2 months, and administration was discontinued after about 1 year. The patient developed sunset glow fundus in the right eye at around disease month 12, but inflammatory findings did not recur. In disease month 26, the corrected visual acuity was 20/16 for the right eye and light perception for the left eye, and no recurrence of inflammatory findings in the right eye or recurrence of corneal epithelial erosion in the left eye has been noted since then. However, at that time, cells floating in the anterior chamber were observed, and the patient was diagnosed with iritis. Although tapering and discontinuation of betamethasone eye drops were attempted, iritis recurred, necessitating continued administration four times a day. Additionally, discontinuation of carteolol 2% eye drops led to an increase in IOP, so administration was continued once daily. Consequently, both betamethasone and carteolol eye drops were maintained. 

In the 34th month of the disease, steroid-induced cataracts developed, and the corrected visual acuity declined to 20/200, leading to cataract surgery. Postoperatively, the patient continued to use betamethasone phosphate eye drops four times a day and carteolol 2% eye drops once a day. At the final visit, 51 months after disease onset and 1 year and 5 months after surgery, the corrected visual acuity was maintained at 20/16. Since then, there has been no recurrence of sympathetic ophthalmia or elevation in IOP. Table 2 summarized the entire clinical course.

**Table 2:** Summary of the clinical course IOP, intraocular pressure

Day	Right eye findings	Left eye findings	Main treatment	Additional treatment
1 (initial visit)		Corneal ulcer	Antibacterial treatment	
41		Corneal perforation and iris prolapse	Therapeutic lamellar keratoplasty	
81		Persistent corneal epithelial defect	Amniotic membrane transplantation	
180	Sympathetic ophthalmia, high IOP (31 mmHg)		Steroid pulse therapy	Carteolol and betamethasone eye drops for the right eye
189		Persistent corneal epithelial defect in the left eye	Conjunctival covering surgery and tarsorrhaphy	Continued carteolol eye drops for the right eye
365	Sunset glow fundus	Conjunctivalization		Continued above treatment
780	Appearance of iritis			Carteolol and betamethasone eye drops for the right eye
810	Resolution of iritis			Continued above treatment
1020	Cataract appearance		Cataract surgery	Continued above treatment

## Discussion

In this case, the mechanism of corneal ulceration remains unclear; however, given that the patient developed a chalazion two months prior, which subsequently ruptured, with persistent tearing and discharge, prolonged inflammation or infection in the eyelid may have been present. Additionally, resistance to treatment following corneal perforation and the presence of a persistent corneal epithelial defect despite amniotic membrane transplantation suggest the possibility of decreased tear secretion as an underlying factor. In most cases, sympathetic ophthalmia occurs because of damage to the uveal tissue in the eye from an ocular perforating injury or during intraocular surgery [1-3], and cases are rare that are not related to either of these causes, i.e., cases involving nontraumatic corneal perforation or not involving intraocular surgery [5,6]. Only a few cases similar to the present one have been reported; in these reports, patients developed sympathetic ophthalmia after fungal keratitis [7] or acanthamoeba keratitis [8]. Tripathy et al. [9] described a case in which the conjunctival flap was created, as a treatment for corneal perforation was incomplete, and contact of the conjunctiva with the iris triggered the development of sympathetic ophthalmia. In the present case, combined conjunctival flap surgery and tarsorrhaphy were performed as a treatment for persistent corneal epithelial defects in the left eye after corneal ulceration and suspicion of infection. Sympathetic ophthalmia was noted immediately after the operation, but it was unlikely to be related to the contact between the conjunctiva and the iris because the period was too short from an immunological perspective. As the remaining possibility, given the previous reports of delayed sympathetic ophthalmia [10,11], we suggest that the damage associated with iris prolapse at the time of the first corneal perforation might have triggered the onset.

TThe estimated overall incidence proportion of sympathetic ophthalmia was 0.19%, as calculated by meta-analysis [12]. The frequency of onset of sympathetic ophthalmia has been reported to be 0.1% to 0.3% after injuries, 0.02% after intraocular surgeries in the United Kingdom and Ireland, and 0.2% to 0.5% after injuries and 0.01% after intraocular surgeries in the United States [1,3]. On the other hand, our previous study showed the frequency was 6.25% after severe open globe injuries that needed emergency surgeries [13]. General clinical findings of sympathetic ophthalmia include corneal precipitates, iritis, vitritis, retinal detachment, and choroiditis in the acute phase and Dalen-Fuchs spots and sunset glow fundus in the chronic phase [1-3]. Extraocular symptoms include neurological symptoms, such as fever, headache, nuchal rigidity, convulsions, and hyperesthesia, and hearing symptoms, such as hearing loss, tinnitus, and ear fullness. These clinical findings and extraocular symptoms are similar to those of VKH, but the frequency of accompanying extraocular symptoms has been reported to be lower in sympathetic ophthalmia [14]. The present patient had no extraocular symptoms. On the other hand, although keratic precipitates, iritis, and serous retinal detachment were noted, increased redness of the optic nerve head was observed. Moreover, FA revealed no multiple hyperfluorescent spots in the early stage and no optic nerve head hyperfluorescence in the late stage, and IA showed no patchy choroidal hypofluorescence. Regarding human leukocyte antigen (HLA), which is considered to be a risk factor for immunological onset due to its role in immune response modulation, we chose not to perform HLA typing for two main reasons. First, the number of individuals who are HLA-DR4 positive is inherently high in Japan, making it less likely to provide decisive diagnostic information. Second, HLA genotyping incurs a high cost of 400 USD for this test alone in Japan, and the patient opted not to proceed due to this expense.

The treatment of sympathetic ophthalmia is the same as that for VKH and basically involves the use of adrenocorticosteroids (steroid drugs) in pulse steroid therapy or high-dose steroid taper. The prognosis is often relatively good if sufficiently high doses of steroids are administered in the early stage of onset; consequently, in recent years, enucleation has been performed much more rarely than in the past [3,15]. In the present case, after conjunctival flap surgery and tarsorrhaphy were performed for persistent corneal epithelial defects, the patient underwent regular follow-up examinations, which enabled the early detection of sympathetic ophthalmia and the initiation of treatment before the condition became severe. The recurrence of sympathetic ophthalmia after treatment with steroids may have occurred because the steroid dose was tapered too quickly; nevertheless, during this time, the patient was visiting the hospital on a weekly basis, leading to early detection, an early increase in steroid dose, and subsequent disappearance of inflammatory findings. After recurrence, the steroid dose was tapered for about 1 year. In the approximately 1 year of follow-up since withdrawal from steroid therapy, the patient has had no recurrence and maintains good corrected visual acuity. However, since signs of iritis were detected at that time, the administration of betamethasone phosphate eye drops was continued. Additionally, as IOP showed a slight increase during this period, treatment with carteolol eye drops was also continued. The elevation in IOP could be attributed to steroid-related side effects and the secondary effects of iritis. However, since the iritis was well-controlled with betamethasone phosphate eye drops at the time of IOP elevation, the former was considered the more likely cause. Furthermore, 10 months after starting betamethasone phosphate eye drops, steroid-induced cataracts developed, necessitating cataract surgery. Although good corrected visual acuity of 1.2 was maintained for 1 year and 5 months postoperatively, careful long-term monitoring is essential after the treatment of sympathetic ophthalmia to check for the recurrence of inflammation and monitor for steroid-induced side effects.

Previous reports have described the occurrence of sympathetic ophthalmia 66 years after injury [11] or 13 years after intraocular surgery [12], suggesting that the patient needs to be followed up in the long term to check for recurrence.

## Conclusions

Therapeutic LKP and amniotic membrane transplantation successfully treated corneal perforation. Six months later, the patient developed sympathetic ophthalmia in the fellow eye, which was managed with prompt steroid therapy and gradual tapering over one year. This case highlights the importance of vigilant follow-up and timely intervention in managing sympathetic ophthalmia.
